# Efficient Treatment of *Sporothrix globosa* Infection Using the Antibody Elicited by Recombinant Phage Nanofibers

**DOI:** 10.3389/fphar.2019.00160

**Published:** 2019-02-27

**Authors:** Feng Chen, Rihua Jiang, Shuai Dong, Bailing Yan

**Affiliations:** ^1^Department of Dermatology, China-Japan Union Hospital of Jilin University, Changchun, China; ^2^Department of Gynecology and Obstetrics, The First Hospital of Jilin University, Changchun, China; ^3^Department of Emergency, The First Hospital of Jilin University, Changchun, China

**Keywords:** sporotrichosis treatment, recombinant phage nanofibers, *Sporothrix globosa*, antibody treatment, immune response

## Abstract

Antifungal therapy is used to treat sporotrichosis. However, there are several limitations in this therapy, such as development of drug resistance and potential health risks including liver injury. The purpose of our study was to evaluate the antifungal efficacy of antibody against the hybrid phage nanofibers displaying KPVQHALLTPLGLDR (phage-KR) in a fungal-infected mouse model. In this study, we extracte an antibody against hybrid phage nanofibers (phage-KR) from immunized mice and passively inoculate *Sporothrix globosa* (*S. globosa*) infected mice. The study shows that the antibody exhibits efficient inhibition efficacy of the *S. globosa* infection, including reduction of the progressive fungi colonizing, increase of animal survival rate and relief of organ inflammation in the mice. The results indicate that antibody against phage-KR may act as a potential strategy for safe and efficient treatment of *S. globosa* infections.

## Introduction

Sporotrichosis is known to be an acute or chronic subcutaneous mycosis, which can affect humans and other mammals (Barros et al., 2011). Especially, the global spread of the cases of sporotrichosis tends to increase in recent years. The disease is usually categorized into four types according to clinical symptoms, including fixed cutaneous, lymphocutaneous, multifocal or disseminated, and extracutaneous (Ramos-e-Silva et al., 2007; Barros et al., 2011). It is believed that *Sporothrix globosa* (*S. globosa*) is the only pathogenic species in northeast China, despite clinical presentations or the regions where it is isolated (Yu et al., 2013).

Currently, antifungal therapy is used to treat sporotrichosis worldwide. However, there are certain limitations in this therapy. Whether administered continuously or intermittently, antifungal treatments can lead to the development of drug resistance in *Sporothrix* (Donnelly et al., 2008). Potential health risks associated with long-term exposure to antifungal agents may cause liver injury in asymptomatic patients, especially in patients with liver disorders, children, and pregnant women (Tuccori et al., 2008). Hence, it is crucial to find an alternative treatment for sporotrichosis, such as antibacterial materials (Lin et al., 2017; Li et al., 2018).

Gp70, a glycoprotein of 70 KDa and a major adhesin expressed on cell surface of *Sporothrix schenckii*, is found to be associated with virulence of fungus (de Almeida et al., 2015; Rodrigues et al., 2015). It plays a key role in immunization modulation and host defense. The monoclonal antibody (mAb) against Gp70 is a candidate for vaccination against sporotrichosis, which may induce strong protection (de Almeida et al., 2015). Four peptides of Gp70 may be involved in this protection. Immunization was confirmed by mass spectrometry of digested Gp70 and the epitopes on the peptides were confirmed by using an epitope-finding algorithm.

**SCHEME 1:**
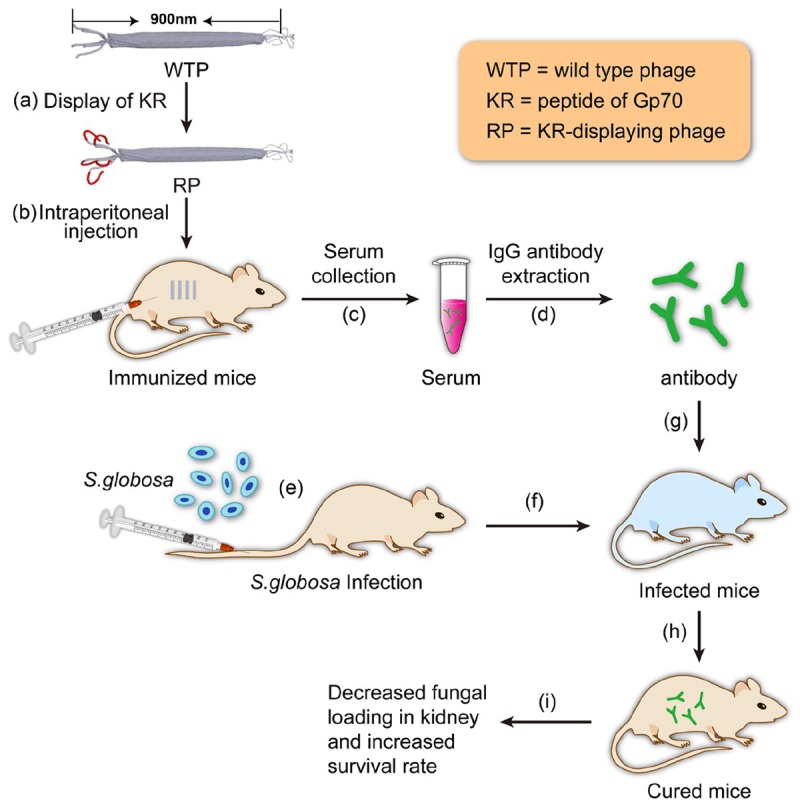
Schematic illustration of using the antibody elicited by RP nanofibers (∼900 nm long and ∼7 nm wide) for treating *Sporothrix globosa* infection.

In our previous studies, it has been found that recombinant phage displaying peptide KPVQHALLTPLGLDR (KR, one of the four peptides) could enhance the immune responses of T helper (Th) 1 and Th17 cells and elicit antibody against Gp70 in BALB/c mice, leading to the inhibition of subsequent infection of the mice (Chen et al., 2017). Phage displaying other three peptides cannot elicit efficient protective immune response. So, in this study, only the antibody against hybrid phage displaying KPVQHALLTPLGLDR (phage-KR) was extracted from the immunized mice and then passively inoculated into *S. globosa*-infected mice. The curing efficacies of the treatment including anti-fungal effects, alleviation of inflammation of the organs and improvement of animal survival rate by the treatment were evaluated (Scheme 1).

## Materials and Methods

### Animals

BALB/c mice (6–8 weeks of age, 20–25 g body weight) were received from Beijing HuaFuKang Biological Technology Co., Ltd. (China). An animal facility with specific pathogen-free conditions was used to raise the mice. All animal procedures in this study were performed in accordance with the Guidelines for Care and Use of Laboratory Animals of Jilin University and approved by the Animal Ethics Committee of The First Hospital of Jilin University (Protocol No. 2017-096-01).

### Strain and Culture Conditions

This study was carried out in accordance with the recommendations of Guidelines for Use of Patient Specimens, Ethics Committee of China-Japan Union Hospital of Jilin University. The protocol was approved by the Ethics Committee of China-Japan Union Hospital of Jilin University. All subjects gave written informed consent in accordance with the Declaration of Helsinki. Cultured isolates were obtained from the patients who were diagnosed with invasive sporotrichosis. Sequence searches in GenBank revealed that all isolates were *S. globosa* strains. The isolates were allowed to grow on Sabouraud dextrose agar slants at 28 °C for 7 days. Fungus was then added to brain heart infusion (BHI) broth and cultured at 37°C for 7 days. The conidia taken from the cultures were diluted to 1 × 10^8^ cells/mL (Lyon et al., 2013; Nunes Mario et al., 2014). The yeast cells were heat-killed for 2 h at 60°C. The heat-killed *S. globosa* (HK-SP) were conserved at 4°C (Tachibana et al., 1999).

### Phages

The sequence of peptide KR was displayed on the gene III of f388-55 phage vector previously. Phage expressing peptide KR could elicit antibody against *S. globosa* and induce a mixed Th1/Th17 response (Supplementary Figure S1). Wild type phages were produced as described previously and conserved in our laboratory (Wang et al., 2014). The phage pellet was allowed to resuspend in PBS.

### SDS-PAGE

Expression of peptide KR by recombinant phage was tested by sodium dodecyl sulfate polyacrylamide gel electrophoresis (SDS-PAGE). The samples of phage were boiled for 10 min in an equal volume of 2× sample loading buffer containing 100 mM Tris–HCl (pH 8.3), 4% SDS, 20% glycerol, and 0.02% bromophenol blue. Proteins were then electrophoresed. The protein bands were shown by silver-staining according to the procedure by Schagger and von Jagow (1987).

### Production of Antibodies

The BALB/c mice were randomly divided into four groups. At a weekly interval, the BALB/c mice were injected intraperitoneally for immunization for four times with different formulations, including 100 μl of PBS containing 25 μg phage-KR nanofibers (denoted as group RP), 100 μl of PBS with 25 μg wild-type phage nanofibers (denoted as group Mock), 100 μl of PBS with 10^8^ HK-SP (denoted as group HK-SP), or PBS only as the negative control (denoted as group PBS). One week after the last immunization, sera were collected from the immunized mice, and IgG antibody was extracted and purified from the sera based on the manufacturer’s procedure by using HiTrap Protein G HP column (a product of GE General Electric, United States).

### Western Blotting

The serum collected from the mice with disseminated sporotrichosis containing antibodies against Gp70 of *S. globosa* or control individuals (de Almeida et al., 2015). The protein was denatured, electrophoresed, and transblotted onto a nitrocellulose membrane in Tris/Glycine buffer. The membrane was blocked in TBS-T with 5% (w/v) non-fat milk at 4°C overnight. Following washing with TBS-T for four times, the nitrocellulose membrane was cultured in a 1:80 dilution of serum in TBST with 5% non-fat milk at 37°C for l h. Following washing, the membrane was further cultured at 37°C with goat anti-mouse IgG conjugated with peroxidase (obtained from Vector Laboratories Inc., of United States) for 1 h, and then stained with 3-amino-9-ethylcarbozole (AEC) for acting as a chromogen.

### Immunofluorescence

1 × 108 sporophores were cultured in 6-well tissue culture plates with RPMI 1640 medium for 4 h. Following slight washing using PBS, the cells were cultured with the sera containing antibodies against recombinant phage displaying peptide KR (RP) or wild type phage (Mock) for 2 h on ice. The adherent cells were fixed using 4% paraformaldehyde for 20 min, followed by washing with PBS three times. The cells were then permeabilized with 0.5% Triton X-100, followed by further washing with TBST three times. After blocking with 3% bovine serum albumin (BSA) at 37°C, the cells were cultured with a 1:1000 dilution of anti-phage g3p (pIII) antibody in PBS buffer (pH 7.4) at 4°C for 1 h, followed by incubation with Cy3 labeled goat-anti-mouse IgG (1:2000) (Abbkine, United States) for another 45 min at 37°C and staining by 4′,6-diamidino-2-phenylindole (DAPI) for 10 min. With washing by PBS containing 0.1% Tween 20 for three times, the cells were viewed by a laser scanning confocal microscope.

### Treating *S. globosa* With Antibodies Against Sporotrichosis

The mouse model infected with disseminated *S. globosa* was established by intravenous injection of 0.2 ml (1 × 10^8^ cells/ml) of *S. globosa* suspension, then the mice were randomly divided into four groups (*n* = 6 per group). One day after infection, mice were intravenously given the following antibodies once every 3 days for a total of three administrations: (1) purified antibody against phage-KR (100 μL); (2) purified antibody against wild-type phage (100 μL); (3) purified antibody against HK-SP (100 μL); and (4) 100 μL PBS. All dosages were 5 mg per kg body weight. Finally, the mice were sacrificed 2 weeks after the final infection.

### Assessment of Protection

To evaluate the colony forming units (CFU) of *S. globosa* at a unit of per gram of tissue, all the kidneys were excised from the mice under aseptic condition (*n* = 6 in each group). The kidneys were weighed and then homogenized in 3 ml of sterile saline by using the glass tissue homogenizers. After that, saline was used to dilute the tissue homogenate, which was then put up on Sabouraud’s dextrose agar. Additionally, for the histological analysis, the kidneys of the mice were removed and fixed with formalin [10% (v/v)]. The buffered paraffin-embedded tissues were sectioned into slices with 3∼5 μm thickness for hematoxylin and eosin (H&E) staining.

To evaluate the survival time of the mice, the mouse model infected with disseminated *S. globosa* was established by intravenous injection of 0.2 ml (5 × 10^8^ cells/ml) of *S. globosa* suspension, and the mice were separated into four groups (*n* = 10 per group). One day post infection the mice were given antibodies intravenously as described above. The survival time was monitored for 2 weeks following treatment.

### Assessment of Liver and Renal Injury

Toxicity was assessed 1 day after passive therapy. Blood samples (50 μl in each mouse) were obtained through the lateral tail vein, followed by transferring into 0.5 mL centrifugation tubes. For each sample, clinical biochemistry parameters were measured by ELISA diagnostic kits (Rongsheng, Shanghai, China; Yutong, Jiangsu, China, respectively) (Chen et al., 2017).

### Statistical Analysis

We examined the differences in the survival time between different groups by the Log-rank test. Analysis of Variance was used for analysis of the data. The criterion for statistical significance was *p* < 0.05.

## Results

### Production of Recombinant Phage

**FIGURE 1:**
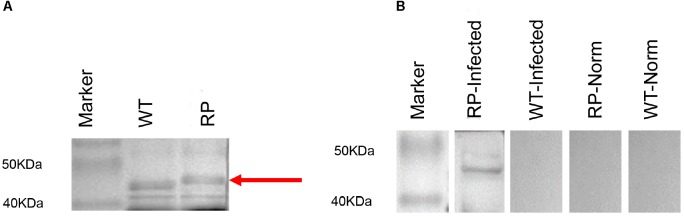
Expression and antigenicity of phage-displayed peptide. **(A)** SDS-PAGE (20%) of wild-type phage (WT) (46 kDa) and recombinant phage (RP) displaying peptide KPVQHALLTPLGLDR. **(B)** Western blot of RP and WT phage with the serum of infected mouse containing an antibody to a 70 kDa protein band, or with the serum of the control individual: Lane 1: Marker; Lane 2: RP phage probed with the serum of infected mouse; Lane 3: WT phage probed with the serum of infected mouse; Lane 4: RP phage with normal serum; Lane 5: WT phage with normal serum.

Expression of phage-KR was evaluated by using SDS-PAGE (Figure 1A). Sera collected from mice infected with systematic sporotrichosis reacted with the fusion protein band in hybrid phage (Figure 1B). The results indicated that the hybrid recombinant phage displayed the peptide KR on the surface.

### Antibody Response Against Phage Displaying Peptide KR in Immunized Mice

**FIGURE 2:**
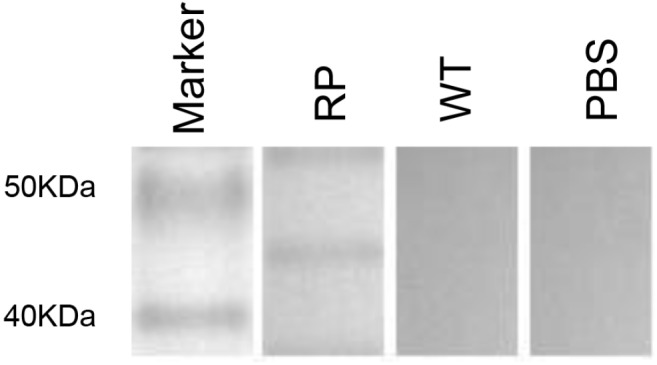
Immunization by using recombinant phage stimulates high levels of KPVQHALLTPLGLDR peptide-specific humoral immunity in mice. Western blot of the sera from recombinant phage-immunized mice with recombinant phage (RP), wild-type phage (WT), or from PBS injection.

**FIGURE 3:**
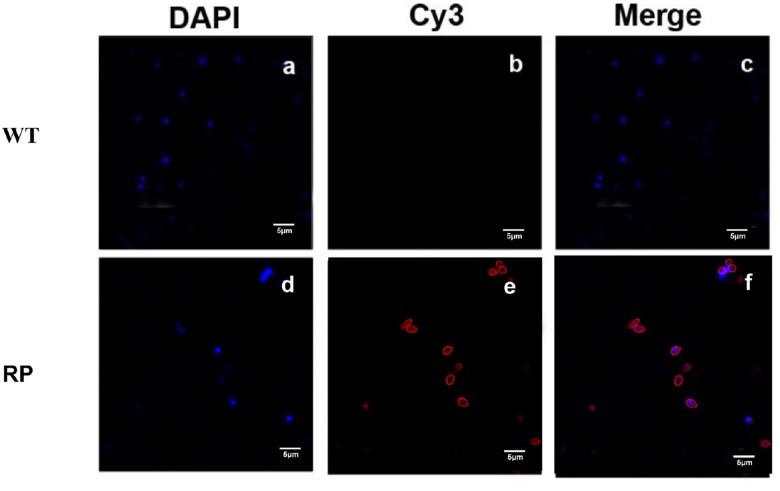
Immunofluorescence to evaluate the binding affinity between the antibody containing sera and *S. globosa*. **(a)** *S. globosa* stained by DAPI (excitation: 358 nm, emission: 461 nm); **(b)** *S. globosa* incubated with the sera containing anti-RP (recombinant phage displaying peptide (KPVQHALLTPLGLDR), followed by staining by Cy3-conjugated goat anti-mouse IgG (excitation: 495 nm, emission: 519 nm); **(c)** Merged picture of image **(a)** and image **(b)**; **(d)** *S. globosa* stained by DAPI (excitation: 358 nm, emission: 461 nm); **(e)** *S. globosa* incubated with anti-WP containing sera, followed by staining with Cy3-conjugated goat anti-mouse IgG (excitation: 550 nm, emission: 570 nm); **(f)** merged picture of image **(d)** and image **(e)**. Recombinant phage (RP); Wild-type phage (WT).

Our results showed that immunization with the hybrid phage expressing peptide KPVQHALLTPLGLDR (phage-KR) produced antibodies in the sera, which is able to bind Gp70 (Figure 2). By immunofluorescence assay, we demonstrated that the antibodies could also recognize Gp70 expressed by *S. globosa* (Figure 3). Hence, the results showed that the recombinant phage displaying peptide KR could exhibit similar function to Gp70 for treatment of *S. globosa* infection collectively. Namely, the peptide KR expressed on the surface of the phage is able to mimic Gp70, a cell surface component, and induce the mice to generate antibodies which can bind to Gp70 for treatment of the infection.

### Assessment of Using Antibody Against Recombinant Phage to Protect Systemic *S. globosa* Infection

**FIGURE 4:**
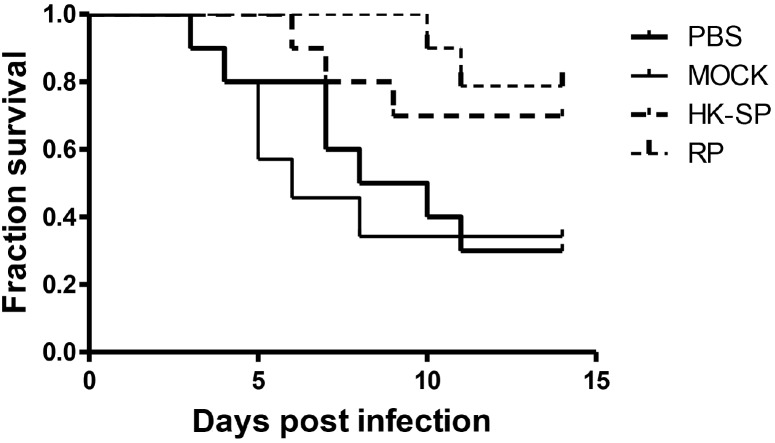
Survival rate of the *S. globosa* infected mice treated with purified antibodies against recombinant phage (RP), wild-type phage (MOCK), or heat-killed *Sporothrix globosa* (HK-SP), or with PBS alone. The mice were intravenously treated once every 3 days for a total of three administrations. All dosages were 5 mg per kg body weight. The group treated with RP vs. PBS-treated group, ^∗^*P* = 0.01; The group treated with HK-SP vs. PBS treated group, *P* = 0.04; Wild-type phage treated group vs. PBS treated group, *P* = 0.79.

**FIGURE 5:**
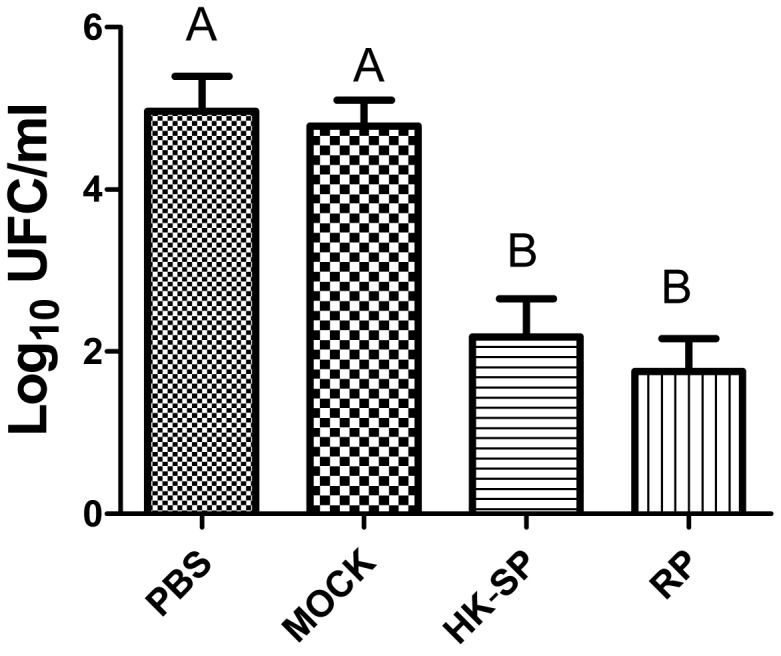
Log CFU/g in the kidneys of the mice. After the mice were sacrificed, and Log CFU/g were quantified 15 days following the immunization. Significantly less CFU in the kidneys of hybrid phage-treated mice was observed than in those treated with wild-type phage (MOCK) and PBS (control). No statistically significant difference between the group treated with HK-SP and that treated with recombinant phage (RP). Values followed by different capital letters differ significantly among the PBS, MOCK, HK-SP, and RP groups.

**FIGURE 6:**
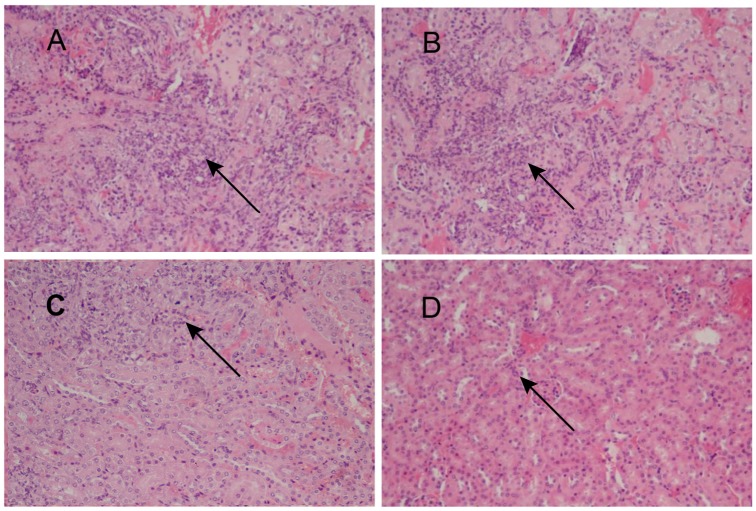
Images of H&E-stained kidney of the *S. globosa*-infected mouse treated with various formulations, Magnification 200×. **(A)** PBS injection **(B)** antibody against wild-type phage injection **(C)** antibody against HK-SP injection **(D)** Antibody against phage-KR injection. **(A,B)** Showed a higher number of inflammatory cells. In contrast **(C,D)** show a dramatic decrease in neutrophil and lymphocyte infiltration. The arrows indicate infiltration of inflammatory cells.

To assess the feasibility of using antibody against Gp70 (anti-Gp70) IgG to treat sporotrichosis, IgG was collected and purified from the sera of the immunized mice, followed by administrating to the mice that were infected with *S. globosa* at a lethal dose (1 × l0^8^ cells). The survival time of the mice was monitored over 14 days (Figure 4). It was found that the group injected with antibody against phage-KR exhibited the highest survival rate (80%). In contrast, the mice treated with PBS showed a much lower survival rate of only 30%. For the group treated with antibody against HK-SP, an increased survival rate (70%) was also found at 14 days following infection. Significantly enhanced survival rate was obtained in the mice treated with antibody against phage-KR compared to the PBS-treated mice. The CFUs in the kidneys of the animals immunized with antibody against phage-KR or antibody against HK-SP were statistically decreased, compared to the mice treated with antibody against wild-type phage or PBS injected mice (Figure 5). The histopathological changes between these groups were consistent with the results of the survival study. Macroscopically, there were no lesions observed in internal organs. However, extensive lymphocytes and neutrophils were observed in the kidney of the group MOCK and group PBS by H&E staining, clearly indicating inflammation. In contrast, only medium levels of lymphocytes and neutrophils were observed in the kidney of the group treated with antibody against phage-KR or antibody against HK-SP (Figure 6).

### Liver and Renal Function

The toxicological assessment of the treatments was carried out. Clinical biochemistry parameters, including aspartate aminotransferase, alanine aminotransferase, alkaline phosphatase, glucose, urea, creatinine, total protein, and albumin, indicated values in normal biological ranges. No obvious difference between the treated groups and control group was observed.

## Discussion

Sporotrichosis is currently distributed throughout the world with a significant increase in human and animal cases over the last two decades. By far, considerable efforts have been made to develop vaccines for human infections caused by *Sporothrix* species. We have previously demonstrated that phage displaying KR could elicit protective immunity against fungal diseases (Chen et al., 2017). In this study, the potential treatment efficacy of anti-phage-KR IgG against Sporotrichosis was explored. We show that anti-HK-SP or anti-phage-KR antibody can reduce the extent of the damage and improve the survival rate of the mice infected with *S. globosa*.

Sporotrichosis is a subcutaneous mycosis resulted from the *S. schenckii* complex, and routine antifungal drugs are not suitable for all patients. Nascimento et al. (2008) demonstrated that the Gp70 molecule is a putative adhesin for fibronectin and laminin, as well as anti-Gp70. Additionally, the yeast cells opsonized with anti-Gp70 mAb increase the phagocytic index (de Almeida et al., 2015). The immunity inhibits interaction between *S. schenckii* yeast cells and the subendothelial matrix.

The humoral immune response appears to prevent and control sporotrichosis infection in mice. Antibodies play an important role in protecting the host from fungal infections, including the agglutination of fungal cells, impeding of fungal attachment, increase of the phagocytosis by host effector cells, neutralizing of immunoregulatory molecules, as well as the complement activation (Nascimento et al., 2008). The incapacity of immune sera to mediate protection from fungi indicates insufficient amounts of protective antibody instead of a fundamental incapability of antibodies in protecting against fungal pathogens. However, phage nanofibers could display peptide KR on the surface through genetic means, allowing the peptide KR to possess a conformation analogical to the native protein. Furthermore, they have the ability to induce the antibody response. Additional benefits include the easiness of production and cost-effectiveness, as well as non-toxicity to humans.

The immunogenicity of epitopes is increased when they are expressed on the phage. Mice sera raised against hybrid phage reacted with peptide KR (Figure 2), suggesting that the specific response of antibody to the hybrid phage may be motivated by peptide KR instead of the components of phage. Antibodies are naturally generated products of the immune system that interact with other immune components. Additionally, protective antibodies may act through the complement-mediated lysis, promotion of phagocytosis, as well as Fc-mediated release of cytokine and direct antimicrobial efficiency (Casadevall et al., 2004). Furthermore, IgG was collocted and purified from the sera of the immunized mice, and then administered into the mice infected with *S. globosa*, leading to significant reduction of the number of CFU and decreased accumulation of inflammatory cells in the kidney. Moreover, injection of IgG significantly enhanced the survival rate of mice infected with *S. globosa*. Our study demonstrates that the antibody against *S. globosa* elicited by phage-KR can efficiently protect the mice from disseminated infection of *S. globosa*. Further studies should examine the protection of phage-KR in other *Sporothrix* strains.

Itraconazole-resistant strains of *S. schenckii* complex have been reported (Oliveira et al., 2011; Ottonelli Stopiglia et al., 2014). The use of phage-KR without adjuvant may lead to significant improvement in the efficacy of antifungal therapy, as well as a reduced inflammation reaction. This approach may avoid the adverse side effects of the antifungal drug, especially for the patients with liver dysfunction, pregnant women, and children, who are not suitable for antifungal drug therapy (Kauffman et al., 2000). It is worth mentioning, although the mechanism for phage responses still remains unknown, no obvious adverse effects of phage have been detected in considerable preclinical studies carried out in various animal models. Although phage-KR would not replace conventional antifungal agents, we show that phage-KR may be an important alternative to existing therapies in some cases (Almeida-Paes et al., 2007).

## Conclusion

In conclusion, this study shows clearly the therapeutic efficiency of the humoral immune response generated by recombinant phage for the treatment of *S. globosa* infection in a fungal infected mouse model. It is notable that the antibody elicited by phage-KR nanofibers displays efficient inhibition of the infection, such as decrease of the progressive fungi colonizing, alleviation of kidney inflammation and reduction of the mortality rate of the mice with sporotrichosis. Overall, the strategy of using antibody against phage-KR can be an efficient and safe approach for the treatment of sporotrichosis infection.

## Data Availability

All datasets generated for this study are included in the manuscript and/or the Supplementary Files.

## Author Contributions

FC, BY, and RJ conceived and designed the experiments and analyzed the data. FC and SD performed the experiments. FC and BY wrote the manuscript.

## Conflict of Interest Statement

The authors declare that the research was conducted in the absence of any commercial or financial relationships that could be construed as a potential conflict of interest.
