# Supplementary material for: Efficient Treatment of Sporothrix globosa Infection Using the Antibody Elicited by Recombinant Phage Nanofibers
Source: Front Pharmacol. 2019 Feb 27;10:160. doi: 10.3389/fphar.2019.00160 (PMC6400886; doi:10.3389/fphar.2019.00160)
Supplement: Supplementary file 1 [file Table_1.DOCX]

**Efficient treatment of *sporothrix globosa* infection using the antibody elicited by recombinant phage nanofibers**

Feng Chen^1^, Rihua Jiang^1,^ *, Shuai Dong^2^, Bailing Yan^3,^ *

^1^Dermatology Department, China-Japan Union Hospital of Jilin University, 126Xiantai Street, Changchun, 130033, China

^2^Department of Gynecology and Obstetrics, The First Hospital of Jilin University, Changchun, 130021, China

^3^Department of Emergency, The First Hospital of Jilin University, Changchun, 130021, China

***Corresponding authors:** Rihua Jiang, 126Xiantai Street, Changchun, 130033, China, [635597795@qq.com](mailto:635597795@qq.com) ; Bailing Yan，The First Hospital of Jilin University, Changchun, 130021,China , [yanbailing@163.com](mailto:yanbailing@163.com).


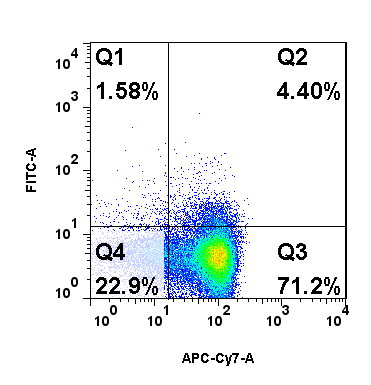


**APC-Cy7-CD4**

**FITC-IFN-γ**


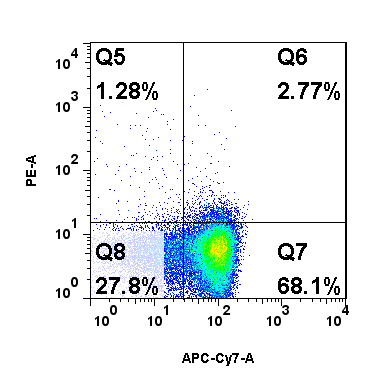


**APC-Cy7-CD4**

**PE-IL-17**


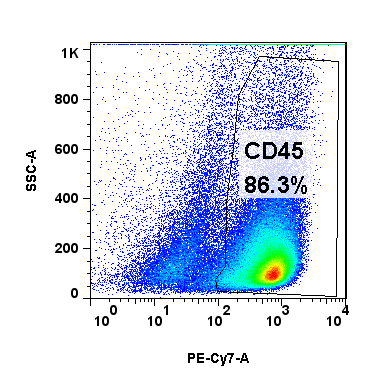


**PE-Cy7-CD45**

**SSC-A**


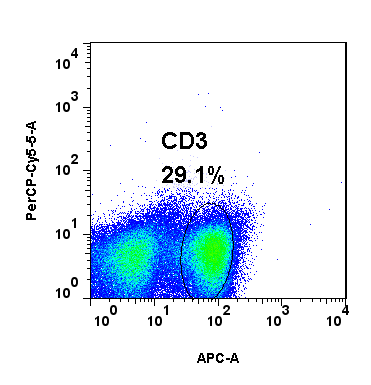


**APC-CD3**

**PercP-Cy5.5**

A

B

C

**Figure S1.** Flow cytometry to analyze populations of Th1 and Th17 cells. (A) Gate strategy (B) The percentages of Th1 cells of the recombinant-phage (RP) group were significantly higher compared to controls (Mock and HK-SP) (*P*< 0. 05)), while that of the Mock and HK-SP did not differ significantly. (C) The percentages of Th17 cells of RP, Mock and HK-SP groups were significantly higher than the PBS controls (*P*< 0. 05), while the RP, Mock and HK-SP groups did not significantly differ. RP: recombinant phage, MOCK: wild-type phage; HK-SG: heat-killed *S. globosa*.
